# Identification and Antioxidant Characterization of Caffeic Acid–Cysteine Adduct in Meat Products Supplemented with Dandelion Extract

**DOI:** 10.3390/foods15101770

**Published:** 2026-05-17

**Authors:** Xiaohan Li, Fengtao Xiang, Shaobing Ye, Yinhong Chen, Hao Sun, Changbo Tang

**Affiliations:** 1 State Key Laboratory of Meat Quality Control and Cultured Meat Development, Key Laboratory of Meat Processing, Ministry of Agriculture, Key Lab of Meat Processing and Quality Control, Ministry of Education, Jiangsu Collaborative Innovation Center of Meat Production and Processing, College of Food Science and Technology, Nanjing Agricultural University, Nanjing 210095, China; 2 College of Sciences, Nanjing Agricultural University, Nanjing 210095, China

**Keywords:** caffeic acid–cysteine adduct, meat products, dandelion extract, antioxidant activity, intestinal absorption

## Abstract

Caffeic acid (CA), a catechol-containing phenolic acid, is readily oxidized to its quinone form during food processing and can form covalent adducts with amino acid residues in proteins. This study aimed to detect the presence of caffeic acid–cysteine adduct (CA-Cys) in meat products supplemented with dandelion extract, and further investigate its antioxidant properties and intestinal absorption characteristics. Compared with CA, CA-Cys exhibited stronger ABTS radical scavenging activity, greater ferric-ion reducing capability, and lower cytotoxicity in cultured cells. In the oxidative stress model induced by hydrogen peroxide (H_2_O_2_) in Caco-2 cells, CA-Cys treatment enhanced antioxidant enzyme activities in a dose-dependent manner. In the Caco-2 cell monolayer model, the apparent permeability coefficient and cellular uptake of CA-Cys were 4.32 × 10^−5^ cm/s and 0.85 ± 0.14 nmol/mg protein, respectively. These values were approximately 1.23-fold and 1.67-fold higher than those of CA, suggesting that CA-Cys may have significant advantages in intestinal absorption. These results indicate that adducts represent potentially beneficial substances for green food processing.

## 1. Introduction

Rising health consciousness among consumers and growing concerns over the potential toxicity associated with synthetic antioxidants, including butylated hydroxytoluene and tertiary butylhydroquinone, have spurred concerns about the utilization of polyphenols in meat products [1]. Notable examples include meat patties enriched with tea polyphenols [2] and sausages supplemented with mulberry polyphenols [3]. Polyphenols containing catechol and pyrogallol groups are easily oxidized to quinones [4]. These quinones can react with nucleophilic amine (-NH_2_) and thiol (-SH) groups via Michael addition reactions in proteins, forming covalent polyphenol–protein adducts [5].

Owing to the kinetic advantage of thiol–quinone reactions over amine–quinone reactions, polyphenols tend to form thiol–phenol adducts with proteins [6], and such adducts have been widely identified and quantified. For instance, Tang et al. [7] identified thiol–polyphenol adducts of rosmarinic acid with cysteine (Cys), glutathione, and hydrolyzed peptide segments of myosin in a Fenton oxidation system. Similarly, myofibrillar proteins can form cysteine–quinone adducts with 4-methylcatechol (4MC) under Fe(II)/H_2_O_2_ treatment [8]. In the presence of light and oxygen, 4MC (1500 ppm) can form adducts with Cys from myofibrillar protein isolate (MPI) at a level of 12.2 nmol/mg MPI in minced beef after 7 days of storage [9]. Additionally, the concentration of chlorogenic acid–Cys adduct can reach up to 52 μM in milk supplemented with 0.1% chlorogenic acid [10]. Although adducts have been extensively identified and characterized in food systems, alkaline models, and Fenton reaction systems, most studies have been conducted using individual polyphenol, which provide clear and definitive insights into their behavior and impacts on food quality. By contrast, real meat systems contain complex polyphenol mixtures and complicated protein environments, and investigations into adduct formation under these more realistic conditions remain comparatively limited.

Modification of polyphenols can also influence the bioactivity of proteins and phenolic acids. Under alkaline conditions, gallic acid can be successfully covalently bonded to soy protein isolate, and the ABTS and DPPH radical scavenging rates of the resulting nanoparticles are significantly increased [11]. Similarly, the adducts formed between myofibrillar protein and chlorogenic acid exhibit better antioxidant activity than chlorogenic acid itself [12]. In addition, adducts formed between chlorogenic acid or caffeic acid (CA) and Cys demonstrated different anti-inflammatory effects from parent phenolic acids in RAW 264.7 cells, with enhanced inhibitory effects on lipopolysaccharide (LPS)-induced inflammatory factors, such as prostaglandin E_2_ (PGE_2_), but their ability to inhibit reactive oxygen species was relatively weak [13].

The stability of bioactive substances during gastrointestinal digestion and their subsequent absorption by small intestinal epithelial cells constitute a prerequisite for their bioavailability [14]. In vitro simulated digestion of adducts showed that the covalent adduct formed by β-lactoglobulin (β-LG) and 4-methylbenzoquinone (4MBQ, the oxidized form of 4MC) releases 1.7 ± 0.1 µmol/g cysteine-4-methylcatechol (Cys-4MC) adducts after digestion, while the remaining Cys adducts exist in peptide-bound forms [15]. This suggests that the adducts released from protein may have the potential to be further absorbed into the bloodstream by small intestinal epithelial cells. However, the capacity of compounds to be absorbed varies greatly with their structures [16], and the intestinal absorption of adducts has not been systematically studied. Therefore, assessing the cytotoxicity, cellular antioxidant activity, and absorption of these adducts is essential for ensuring the safe and effective use of polyphenol-modified meat products.

Dandelion extract represents a valuable medicinal and edible homologous resource rich in phenolic acids and flavonoids (such as CA, rutin, luteolin 7-glucoside) [17,18]. Notably, CA, with its simple catechol structure, can substantially affect food quality by forming covalent adducts with thiol-containing proteins [19,20]. Furthermore, Liu et al. (2023) [13] have shown that the bioactivity of CA is remarkably modified as a result of cysteine adduct formation. It is of notable importance in food matrices. Herein, we aimed to identify the formation of CA-Cys adduct in meat patties supplemented with CA-rich dandelion extract. Additionally, we evaluated how effectively the CA-Cys adduct exhibited antioxidant activity, utilizing both chemical assays and cell models. Finally, the intestinal absorption and cellular uptake of the CA-Cys adduct were assessed using a Caco-2 cell monolayer model. This approach aims to preliminarily elucidate the functional properties of CA-Cys adduct in polyphenol-modified meat products, providing insights into their potential as a bioactive ingredient.

## 2. Materials and Methods

### 2.1. Materials

Pork longissimus muscle was obtained from a Shuanghui fresh meat store (Nanjing, China). The compound phosphate was purchased from Xuzhou Tian’an Food Technology Co., Ltd. (Xuzhou, China). Dandelion extract (10:1 ethanol extraction), fluorescein disodium salt (S19118), alkaline phosphatase (ALP) kit (R21783), glutathione peroxidase (GSH-Px) kit (R21876), and CA (≥98%) were sourced from Yuanye (Shanghai, China). Cys (≥99%), Amberlyst A26 hydroxide resin, and Amberlyst A21 resin were purchased from Macklin (Shanghai, China). Periodic acid, tetrahydrofuran (≥99%), and methyl tert-butyl ether (≥99%) were supplied by Titan (Shanghai, China). Pepsin (P6887), Pronase E (P5147), and fetal bovine serum were sourced from Sigma-Aldrich (Shanghai, China). Catalase (CAT) kit (BC0205) was purchased from Solarbio (Beijing, China). Cell Counting Kit-8 (CCK-8), Superoxide dismutase (SOD) assay kit (S0109) was obtained from Beyotime (Shanghai, China). The human colon adenocarcinoma cell line (Caco-2) was purchased from Wuxi Dudi Biotechnology Co., Ltd. (Wuxi, China).

### 2.2. Analysis of Phenolic Compounds in Dandelion Extract

Analysis of dandelion extract was conducted on the ExionLC system (Shimadzu, Tokyo, Japan). The MS analysis was performed on a TripleTOF 5600+ (AB SCIEX, Marlborough, MA, USA) spectrometer. The UPLC separation program is provided in Table S1. MS parameters (ESI + mode, mass range 50–1600 *m*/*z*) are provided in Table S2.

### 2.3. Synthesis of CA-Cys

CA-Cys adduct was synthesized as described by Poojary et al. [10], with modifications. Periodic acid (6 g) was dissolved in water (30 mL) with Amberlyst A26 resin hydroxide (10.8 g), followed by stirring for 2 h. The resin was isolated and washed repeatedly: four times with water, twice with tetrahydrofuran, and a final rinse with methyl tert-butyl ether. The obtained residue was dried overnight at 35 °C in a vacuum, yielding 8.08 g of oxidized resin. The synthesis reaction was carried out in batches. In a representative procedure, 1.5 g of oxidized resin was added to the solution of CA (292 mg) in acetonitrile/dimethylformamide (4:1 *v*/*v*, 32 mL) and maintained under stirring for 20 min. The resin was subsequently removed via filtration, and the resulting filtrate was transferred to phosphate buffer (32 mL, 0.1 M, pH 6.5) supplemented with 196.2 mg of cysteine. After stirring the reaction mixture overnight, an AONUO LC2100 preparative HPLC system was used to purify the sample. Real-time monitoring of chromatography was used to collect target components with desirable purity, which were then combined and lyophilized. The resulting product was dissolved in water and treated with Amberlyst A21 resin overnight to remove the trifluoroacetic acid salt, followed by filtration using a G4 sand-core funnel (pore size of 3–4 μm) at 25 °C and lyophilization to obtain the final solid product (99.15% purity).

For structural characterization, the ^1^H nuclear magnetic resonance (NMR) spectrum of the CA-Cys adduct was collected in deuterated dimethyl sulfoxide (DMSO-*d*_6_) using a Bruker-DRX 400 MHz spectrometer (BRUKER, Berlin, Germany). LC-MS/MS analysis of CA-Cys standard was determined by the ACQUITY UPLC I Class system (Waters, Milford, MA, USA). The MS analysis was performed on the ZenoTOF^TM^ 7600 (AB SCIEX) mass spectrometer. Table S3 presents the separation program, and mass spectrometry parameters (ESI+ mode, mass range 50–1000 *m*/*z*) are shown in Table S2.

### 2.4. Preparation of Pork Patties

Five experimental groups were established, including a blank control (no dandelion extract or CA), a positive control (0.1% CA, *w*/*w*), and three treatment groups (0.5%, 1%, or 2% dandelion extract, *w*/*w*). All supplementation ratios were calculated based on the weight of 250 g minced pork. Dandelion extract and caffeic acid were separately dissolved in 20 mL of composite phosphate solution, and the mixture was constantly stirred for 4 h at 4 °C. Composite phosphate was added at 0.4% (*w*/*w*) of meat. 3.75 g of salt, 1.25 g of sugar, and polyphenol solutions (4 °C) were homogenized with 250 g of minced pork. The mixture was chopped for 3 min, paused for 2 min, and repeated twice. Ice (20 g) was then added and chopped for 2 min. The mixture was then marinated at 4 °C for 12 h and processed into patties (6 × 1.5 cm), which were baked (180 °C, 20 min) and stored at −80 °C.

### 2.5. Identification of CA-Cys Adduct in Pork Patties

Protein was isolated from the pork patties according to Zhou et al. [21], with minor modifications. Briefly, samples were homogenized at 7000 rpm for 2 × 30 s with four volumes of extraction buffer (pH 7.0) containing 0.1 M KCl, 2 mM MgCl_2_, 20 mM K_2_HPO_4_/KH_2_PO_4,_ and 1 mM EGTA, followed by centrifugation at 2000× *g* for 10 min. This extraction procedure was performed in duplicate. The resulting pellet was washed twice with 0.1 M KCl at a ratio of 1:4 (*w*/*v*), and the final protein pellet was collected for subsequent analysis. Protein hydrolysis was performed with modifications from Krax et al. and Poojary et al. [22,23]. A total of 4 mg of protein was mixed with 1 mL of 0.02 M hydrochloric acid, vortexed, and incubated at 4 °C for 2 h. Subsequently, pepsin (600 U/sample) was added to the mixture, followed by incubation at 37 °C with shaking (180 rpm) for 24 h to allow hydrolysis. Subsequently, 300 μL of borate buffer (800 mM) and 50 μL of methanol were added and adjusted to pH 7.0 using 1 M NaOH. After the addition of Pronase E solution (4 U/sample), the reaction was maintained at 50 °C for 24 h. Finally, the mixture was centrifuged at 12,000 rpm for 10 min, and the supernatant was passed through a 0.22 μm filter. Mass spectrometric detection of CA-Cys adduct was carried out on the Triple Quad 6500+ mass spectrometer (AB SCIEX). The chromatographic separation program is detailed in Table S4, while the mass spectrometry parameters (ESI+ mode, mass range 50–1000 *m*/*z*) are summarized in Table S2, with collision energy adjusted to 45 V.

### 2.6. Antioxidant Activity of CA-Cys Adduct

#### 2.6.1. DPPH Radical Scavenging Activity

Briefly, 50 μL of CA or CA-Cys solution at concentrations of 10, 25, 50, and 100 μM was mixed with DPPH solution (150 μL, 0.1 mM, in 95% ethanol). Trolox was utilized for comparative purposes as a positive control. The mixture was incubated in the dark for 30 min, and the absorbance (517 nm) was measured using a Spectra Max M2 microplate reader (MD, San Jose, CA, USA). Calculations followed the protocol described by Zhou et al. [24].

#### 2.6.2. ABTS and Total Antioxidant Capacity (T-AOC) Assays

ABTS solution preparation and result calculation followed the protocol reported by de Camargo et al. [25]. Briefly, ABTS solution (0.2 mL) was mixed with 20 μL of CA or CA-Cys solution at concentrations of 50, 100, 200, and 400 μM. After incubation in the dark for 6 min, the absorbance of the mixture was measured at 734 nm using a microplate reader. T-AOC (100–400 μM) was assayed following the instructions of the T-AOC kit (BC1310, Solarbio, Beijing, China). Trolox was used as a positive control for comparison.

### 2.7. Cell Viability

The Caco-2 cells were maintained in Dulbecco’s Modified Eagle Medium (DMEM) containing 10% (*v*/*v*) of fetal bovine serum (FBS, Sigma, Saint Louis, MO, USA) and 1% penicillin–streptomycin (*v*/*v*) with incubation settings at 37 °C and 5% CO_2_. Briefly, after cell (1 × 10^4^ cells per well) attachment, cells were treated with 100 μL of CA and CA-Cys (0, 150, 300, 600, and 900 μM, 0.3% DMSO) for 24 h. Finally, the culture medium was replaced with 110 μL of CCK8 solution (DMEM: CCK8 = 10:1) for another 2 h. Detection was performed using a Spectra Max M2 microplate reader (450 nm). Furthermore, the cytotoxic impact of H_2_O_2_ (0–1.75 mM) on the Caco-2 cells was determined under similar conditions, using a 4 h H_2_O_2_ incubation period.

### 2.8. Cellular Antioxidant Activity Assay

After cells’ (5 × 10^4^ cells per well) attachment in 12-well plates, the cells were exposed to CA-Cys and CA (0, 50, 100, and 300 μM, 0.1% DMSO) for 24 h and washed twice. The control group was treated with DMEM, and cells in each experimental group were further exposed to 1 mM H_2_O_2_ for 4 h. Finally, the cells were subjected to ultrasonication in ice water (200 W, 3 s on and 10 s off, 20 cycles) for lysis. The resulting supernatant was collected following centrifugation of the lysate (8000× *g*, 10 min, 4 °C). Commercial assay kits enabled the measurement of the enzymatic activities of SOD, CAT, and GSH-Px within the samples.

### 2.9. Establishment of the Caco-2 Cell Monolayers

Permeability assays followed the method of Chen et al. [14], with modifications. 5 × 10^4^ cells per well of the Caco-2 cells were plated on top of the polycarbonate membrane to establish a Caco-2 cell monolayer on the transwell plate (1.12 cm^2^ membrane area). These cells were cultured for three weeks to ensure that confluent and tight monolayers were formed. Transepithelial electrical resistance (TEER) was measured on the 3rd, 7th, 11th, 15th, 19th, and 21st days using a volt ohmmeter (Millicell-ERS-2, Merck KGaA, Boston, MA, USA). To confirm the tight junctions, the cell monolayers were incubated with fluorescein disodium salt (25 μg/mL) for 120 min to evaluate the apparent permeability coefficient (*P*_app_). The culture supernatant was collected for testing alkaline phosphatase (ALP) activity. The following equation was employed for the calculation of TEER values:

(1) $$TEER\left(\Omega \cdot {cm}^{2}\right)=\left(\Omega_{sample}-\Omega_{blank}\right)\times 1.12$$

where Ω*_sample_* and Ω*_blank_* denote the TEER value of the sample and the blank wells, respectively, and 1.12 refers to the effective membrane area (cm^2^).

### 2.10. Transport and Cellular Uptake

Before the experiment, the monolayers were washed and balanced twice with Hank’s Balanced Salt Solution (HBSS, Biosharp, Hefei, China). Subsequently, the HBSS on both sides was replaced by the following solution: CA-Cys or CA (300 μM, 0.5 mL) was added to the apical (AP) chamber, while 1.5 mL of HBSS was added the basolateral (BL) chamber. Subsequently, the culture plates were maintained in a 5% CO_2_ incubator at 37 °C for 2 h, and the samples were collected from the BL side. Finally, after being washed twice with HBSS, the Caco-2 monolayers were lysed with RIPA buffer. The lysates were centrifuged, and the content of CA-Cys or CA in the supernatants was analyzed according to Section 2.5. Calculation of *P*_app_ proceeds as follows:

(2) $$P_{app}=\frac{dQ/dt}{A\times C_{0}}$$

where d*Q*/d*t* (µg/s) represents the transport amount during the experiment. *A* denotes the effective membrane area (cm^2^), and *C*_0_ refers to the sample initial concentration.

### 2.11. Statistical Analysis

GraphPad Prism (version 10.6) and SPSS software (version 20.0) were used for image and statistical analysis. Two treatment groups were compared using the *t*-test. More than two groups were compared using one-way analysis of variance with Duncan’s multiple comparison test. All data were presented as mean ± SD of at least triplicate measurements. *p* < 0.05 was considered statistically significant.

## 3. Results

### 3.1. Identification of Phenolic Compounds in Dandelion Extract

By comparing theoretical and observed mass-to-charge ratios (*m*/*z*) and in combination with the relevant literature reports, nine major phenolic compounds were identified in total (Table 1), including five phenolic acids (protocatechuic acid, caftaric acid, caffeic acid, 4-hydroxybenzoic acid, chlorogenic acid), two flavonols (rutin, kaempferol-3-O-rutinoside), one flavone (cynaroside), and one coumarin (esculin).

### 3.2. Structural Characterization of CA-Cys

**Figure 1:**
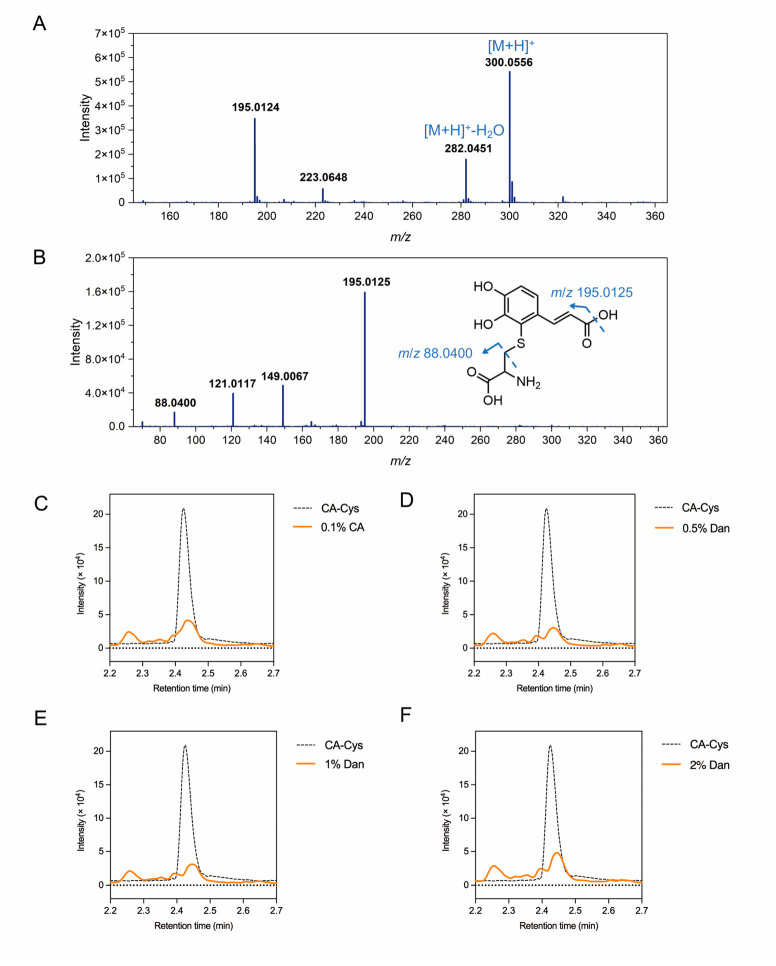
Structural characterization of CA-Cys standard and identification of CA-Cys in meat patties. (**A**) MS and (**B**) MS/MS spectra of CA-Cys standard, XIC chromatograms (*m*/*z* 300.1/195.0) of meat patties supplemented with (**C**) 0.1% CA, (**D**) 0.5% Dan, (**E**) 1% Dan, and (**F**) 2% Dan. The XIC chromatograms represent the mean values of experimental replicates (*n* = 3).

CA-Cys adduct was synthesized and characterized using UPLC–MS/MS to to further verifies the presence of CA-Cys adduct in meat samples. CA-Cys was isolated through chromatography under ESI positive ion mode, producing [M + H]^+^ ions in the mass spectrometer. By extracting the ion current based on the theoretical *m*/*z*, the ion peak of CA-Cys (C_12_H_13_NO_6_S, [M + H]^+^ = 300.0556) and its dehydrated ion peak ([M + H]^+^ − H_2_O = 282.0451) were obtained in the mass spectrum (Figure 1A). MS/MS analysis of the precursor ion at *m*/*z* = 300.0556 generated characteristic fragment ions at *m*/*z* = 88.0400 and *m*/*z* = 195.0125 (Figure 1B), matching the ion peak formed from the C–S bond cleavage of cysteine and the ion peak of CA with a sulfur residue after the loss of one hydroxyl group [10]. We also observed fragments with *m*/*z* = 121.0117 (C_7_H_5_S^+^) and *m*/*z* = 149.0067 (C_8_H_5_OS^+^), as reported by Krax et al. [22], which correspond to a sulfur atom bonded to the first side-chain carbon of CA after the benzene ring loses two hydroxyl groups, and sulfur cyclizing with the second side-chain carbon of CA after losing one hydroxyl group. NMR analysis further verified the molecular structure of the adduct (Figure S1). ^1^H NMR (400 MHz, DMSO-*d*_6_) δ 8.22 (d, J = 15.9 Hz, 1H), 7.23 (d, J = 8.4 Hz, 1H), 6.80 (d, J = 8.3 Hz, 1H), 6.30 (d, J = 15.9 Hz, 1H), 3.01 (t, J = 7.5 Hz, 1H), 2.82 (d, J = 7.5 Hz, 2H). ^1^H NMR spectra revealed two aromatic doublets at δ 7.23 and 6.80 with a coupling constant of approximately 8.4 Hz. This characteristic ortho-coupling indicates that the substitution occurred at the C-2 position, placing the H-5 and H-6 protons in an ortho-relationship. The chemical shifts and the characteristic splitting pattern of the aromatic protons were found to be identical to the C-2 adduct described by Krax et al. [22].

### 3.3. Identification of CA-Cys Adduct in Meat Patties

The fragment ion at *m*/*z* = 195.0 was chosen as the qualitative ion for CA-Cys from the product ions shown in Figure 1B, owing to its highest relative abundance. Subsequently, the protein-derived CA-Cys adduct in the meat patty samples were detected through triple quadrupole mass spectrometry in the MRM mode, which provides high sensitivity and selectivity and is appropriate for detecting target molecules in complex samples [26]. The extracted ion chromatogram (XIC) of CA-Cys is shown and a single peak appeared at 2.43 min. In the XIC of hydrolyzed pork patty samples supplemented with CA and dandelion extract, mass spectrometry peaks corresponding to CA-Cys were also observed (Figure 1C–F), indicating that CA covalently reacted with the Cys residues of meat proteins. The formation of these adducts likely involves a synergistic mechanism driven by the processing conditions. Composite phosphates facilitate the autoxidation of CA, converting it into reactive quinone. Concurrently, the presence of phosphates increased the ionic strength of the meat system, promoting the dissociation of actomyosin and the protein solubilization. Relative to other nucleophilic groups of proteins, the thiol group exhibits a distinct kinetic preference and is highly reactive toward quinones [27]. T Arsad et al. [28] reported that the swift formation of adducts (Cys-4MC) in raw beef systems is swift even at the early stages of 4MC supplementation. Additionally, heating may alter the conformation of protein molecules, exposing the originally hidden reactive thiol groups [29]. Some of these reduced thiol groups are oxidized to form disulfide bonds, while others react with quinones to form thiol–quinone adducts [30].

### 3.4. Antioxidant Properties of CA-Cys and CA

**Figure 2:**
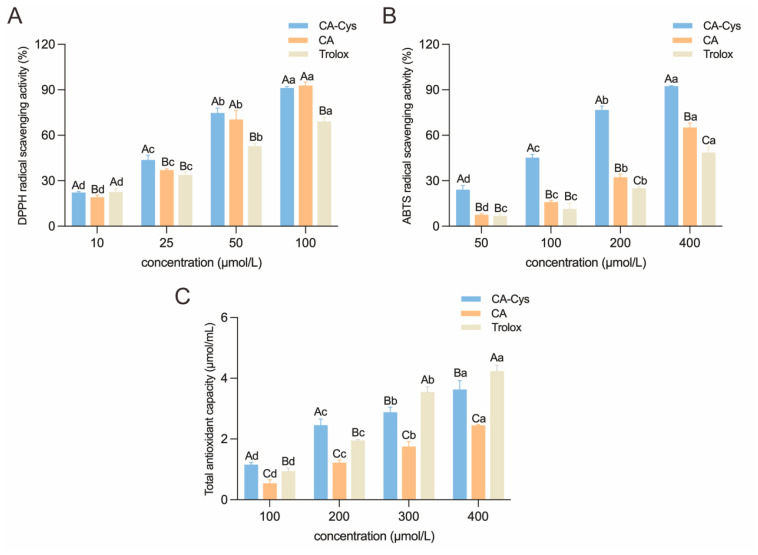
Determination of the antioxidant capacities of CA-Cys and CA in different antioxidant assays. (**A**) DPPH radical scavenging activity, (**B**) ABTS radical scavenging activity, (**C**) total antioxidant capacity. Data are presented as mean ± SD (*n* = 3). Different lowercase letters indicate significant differences within groups (*p* < 0.05). Different capital letters indicate significant differences between groups (*p* < 0.05).

Polyphenols with catechol groups can exert antioxidant activity by scavenging free radicals through hydrogen atom transfer by phenolic hydroxyl groups [31]. Both CA-Cys and CA have a catechol structure; the results showed that covalent binding with Cys significantly enhanced the antioxidant capacities of CA. CA-Cys exhibited an enhanced DPPH scavenging ability as the concentration increased. When the concentration was 100 μM, the scavenging rates of CA-Cys, CA, and Trolox were 91.27%, 92.83%, and 69.12%, respectively (Figure 2A). At 400 μM, the ABTS clearance rate of CA-Cys was 92.48% and that of CA was 65.11% (*p* < 0.05, Figure 2B). Similarly, the T-AOC of CA-Cys was 3.63 ± 0.29 µmol/mL, 1.48-fold higher than that of CA (*p* < 0.05, Figure 2C). Miura et al. [32] indicated that in meat products, 2′-S-cysteinylcaffeic acid showed greater ability to reduce metmyoglobin than CA or Cys alone. Meanwhile, the substitution of cysteine groups effectively modulates the potential pro-oxidant side effects of CA, thereby maintaining the ideal red color of fresh meat. As previous studies have shown, covalent modification of cysteine can enhance hydrogen supply capacity by reducing the bond dissociation energies of phenolic hydroxyl O-H bonds, which is related to the conjugative effect produced by the sulfur atom [33]. The antioxidant capacity of polyphenols exhibits a significant negative correlation with the O-H bond dissociation energy of phenolic hydroxyl groups. The lower O-H bond dissociation energy corresponds to the stronger antioxidant capacity exerted by the compound via the hydrogen atom transfer (HAT) mechanism [34].

### 3.5. Cellular Antioxidant Activity of CA-Cys and CA

#### 3.5.1. Cell Viability Analysis

**Figure 3:**
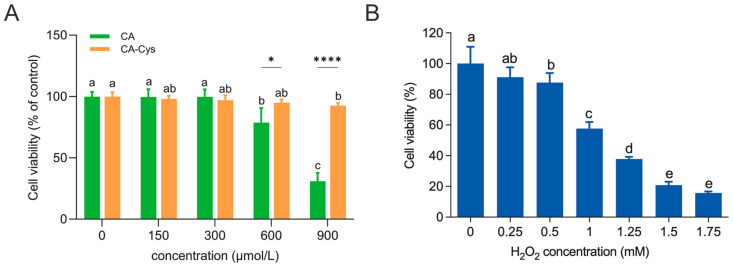
Caco-2 cell viability. (**A**) Cell viability following CA-Cys and CA at different concentrations. (**B**) Cell viability of Caco-2 cells induced by H_2_O_2_. Data were shown as mean ± SD from independent experiments (*n* = 3). Different lowercase letters indicate significant differences within groups (*p* < 0.05). * *p* < 0.05 and **** *p* < 0.0001 compared with comparison group.

To investigate the cytotoxicity of CA-Cys and CA, we exposed Caco-2 cells to different doses of CA-Cys or CA (0, 150, 300, 600, 900 μM) for 24 h. At these concentrations, Caco-2 cells treated with CA-Cys maintained a viability rate above 90%, showing no obvious cytotoxicity (Figure 3A). Conversely, a marked reduction in cells was observed at high doses of CA. At CA concentrations of 600 and 900 μM, the cell viability decreased significantly to 78.66% and 30.97%, respectively (*p* < 0.05). Concentrations below 300 μM were chosen for subsequent experiments comparing the protective effects of CA-Cys and CA against cellular oxidative stress.

Figure 3B depicts that H_2_O_2_ induced damage to Caco-2 cell viability with increasing concentrations. When exposed to 1 mM H_2_O_2_, the cell viability decreased to 57.68% (*p* < 0.05). This concentration is close to the optimal oxidative stress threshold (50% viability) and will not result in excessive cellular damage [35]. Therefore, in subsequent experiments, 1 mM H_2_O_2_ was applied to Caco-2 cells to establish an oxidative stress model.

#### 3.5.2. Cellular Antioxidant Activity

**Figure 4:**
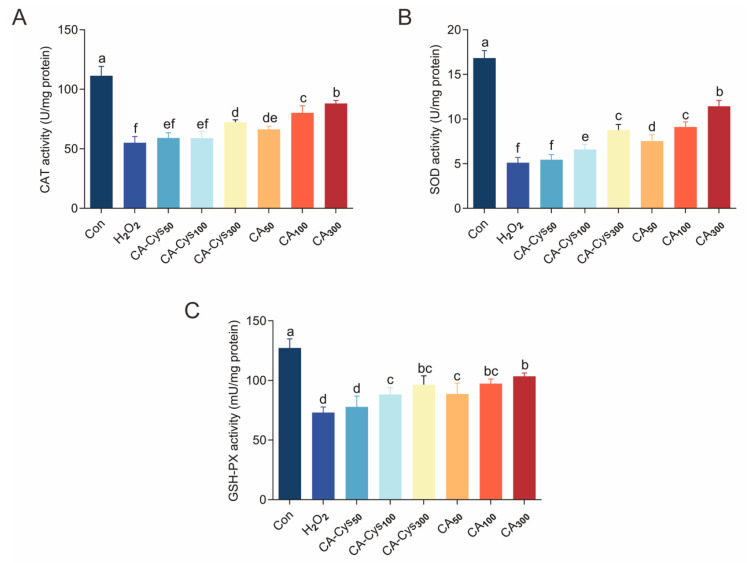
Influence of CA-Cys and CA on H_2_O_2_-induced Caco-2 cells. (**A**) CAT activity; (**B**) SOD activity; (**C**) GSH-Px activity. Data were shown as the mean ± SD (*n* = 4). CA_50_, CA_100_, and CA_300_ represent Caco-2 with CA in 50 μM, 100 μM, 300 μM, respectively. CA-Cys_50_, CA-Cys_100_, and CA-Cys represent Caco-2 with CA-Cys in 50 μM, 100 μM, 300 μM, respectively. Different lowercase letters indicate significant differences between groups (*p* < 0.05).

Antioxidant enzymes are the main components of the cellular antioxidant defense system and can maintain redox balance by eliminating excessive free radicals [36]. The beneficial regulation of CA-Cys and CA on the endogenous antioxidant defense system is revealed in Figure 4A–C. Compared with the control group, the H_2_O_2_ treatment group exhibited a significant decrease in antioxidant enzyme activity (*p* < 0.05). However, relative to the model group, the CAT activities in cells pretreated with CA-Cys were raised to 131.14%, and those in the CA group reached 159.80% at a treatment dose of 300 μM (Figure 4A). This indicates that the CA-Cys and CA have a positive regulatory effect on the activity of antioxidant enzymes. At a treatment dose of 300 μM, the CA-Cys and CA groups did not differ significantly in GSH-Px enzyme activity (Figure 4C, *p* > 0.05). Similarly, Li et al. [37] reported that *Isodon serra* polyphenol (rabdosiin) can increase the activity of SOD enzymes in t-BHP-induced HepG2 oxidative stress, and Bernatoniene and Kopustinskiene [38] further elucidated the mechanism by which catechins alleviate cellular oxidative stress through the combined effects of direct actions (scavenging reactive oxygen species) and indirect actions (inducing antioxidant enzymes).

### 3.6. Verification of Caco-2 Cell Monolayers

**Figure 5:**
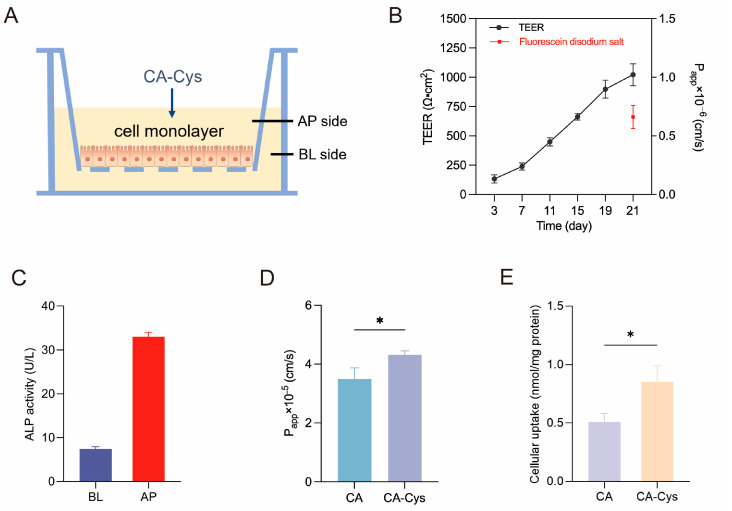
Establishment of the Caco-2 cell monolayer model and absorption of CA-Cys and CA. (**A**) Caco-2 cell monolayer model; (**B**) TEER value of Caco-2 cell monolayers and *P*_app_ value of Fluorescein disodium salt; (**C**) ALP activity on both sides of the 21st day; (**D**) *P*_app_ value of CA-Cys and CA; (**E**) cellular uptake of CA-Cys and CA in Caco-2 model. Data are presented as the mean ± SD (*n* ≥ 3). * *p* < 0.05 compared with the comparison group.

Upon reaching confluence, Caco-2 cells start to differentiate and form tightly connected monolayers [39]. Their polarity distribution, transport system, and expression of specific enzymes are highly analogous to those in small intestinal epithelial cells [40], making them widely applicable as model systems to predict the uptake and absorption of active components in the intestine. The successful construction of the model is typically verified by determining the TEER, ALP activity, and fluorescein disodium salt permeability [41]. Our results indicate a gradual increase in TEER with culture time (Figure 5B). On the 21st day, the TEER value exceeded 800 Ω·cm^2^, and the *P*_app_ of fluorescein disodium salt was 0.66 × 10^−6^ cm/s (Figure 5B), indicating that the cell monolayers were dense and their structure was intact [42]. ALP, a brush margin marker enzyme generated during the monolayer differentiation of Caco-2 cells, gradually rises throughout the culture process and is primarily concentrated on the AP side [43]. The ALP activity ratio between the AP and BL sides of the monolayer indicates the level of cell polarization [44]. On the 21st day of culture, the BL side exhibited significantly lower ALP levels compared to the AP side (*p* < 0.05, Figure 5C), indicating that membrane differentiation and cell polarization was accomplished, allowing for the next step of the experiment.

### 3.7. Transport and Uptake of CA-Cys and CA

To investigate the cellular uptake and *P*_app_ value of CA-Cys and CA, a 300 μM sample solution was added to the top side. The *P*_app_ value is regarded as a critical indicator of the Caco-2 monolayer model, because it reflects the intestinal absorptive potential of a compound [45]. When the *P*_app_ value from the AP → BL side is greater than 1 × 10^−5^ cm/s, this indicates that a compound has high permeability [42]. Figure 5D shows that the *P*_app_ value of CA-Cys was 4.32 × 10^−5^ cm/s, higher than that of CA at 3.50 × 10^−5^ cm/s (*p* < 0.05), indicating that CA-Cys and CA are well absorbed in the small intestine. This experiment also analyzed the contents of CA-Cys and CA in the cells at 120 min. The cellular uptake of CA-Cys was 0.85 ± 0.14 nmol/mg protein, 1.67-fold higher than that of CA, indicating that the cellular accumulation of CA-Cys is more significant (*p* < 0.05, Figure 5E). These results indicate that CA-Cys has the potential for intestinal permeability.

## 4. Conclusions

In conclusion, we detected CA-Cys in meat products supplemented with dandelion extract and initially investigated the antioxidant, transport, and uptake capabilities of CA-Cys. The results indicated that CA-Cys had significant free radical scavenging ability and lower cytotoxicity than CA. It could effectively alleviate cellular oxidative stress damage through upregulating antioxidant enzyme activity. In addition, the transport capacity of CA-Cys in Caco-2 cell monolayers was superior to that of CA. Subsequent studies can combine in vitro simulated gastrointestinal digestion models to analyze the release and digestive stability of CA-Cys in protein matrices. Moreover, considering the protective mucus layer and the metabolic effects of intestinal flora on polyphenols, future research can further explore the absorption and metabolism mechanism of the adducts. These findings suggest that phenolic–amino acid or protein adducts such as CA-Cys formed during green food processing may represent promising functional ingredients with effective antioxidant properties.

## Figures and Tables

**Table 1:** Phenolic compounds identified in dandelion extracts by UPLC-MS/MS.

No.	Compound	Formula	tR/min	Mass (Da)	Adduct	Extraction Mass (Da)	Found at Mass (Da)	Error (ppm)
1	Protocatechuic acid	C_7_H_6_O_4_	4.53	154.02661	+H	155.03389	155.03384	−0.3
2	Caftaric acid	C_13_H_12_O_9_	4.72	312.04813	+H	313.05541	313.05560	0.6
3	Caffeic acid	C_9_H_8_O_4_	4.95	180.04226	+H	181.04954	181.04931	−1.2
4	Esculin	C_15_H_16_O_9_	5.21	340.07943	+H	341.08671	341.08764	2.7
5	4-hydroxybenzoic acid	C_7_H_6_O_3_	5.26	138.03169	+H	139.03897	139.03923	1.9
6	Chlorogenic acid	C_16_H_18_O_9_	5.64	354.09508	+H	355.10236	355.10208	−0.8
7	Rutin	C_27_H_30_O_16_	7.01	610.15339	+H	611.16066	611.16378	5.1
8	Kaempferol-3-O-rutinoside	C_27_H_30_O_15_	7.51	594.15847	+H	595.16575	595.16579	0.1
9	Cynaroside	C_21_H_20_O_11_	7.73	448.10056	+H	449.10784	449.10776	−0.2

## Data Availability

The original contributions presented in the study are included in the article and Supplementary Materials; further inquiries can be directed to the corresponding author.

## Supplementary Materials

The following supporting information can be downloaded at: https://www.mdpi.com/article/10.3390/foods15101770/s1, Figure S1: ^1^H NMR spectrum of CA-Cys, Table S1: The gradient elution program for dandelion extract, Table S2: The mass spectrometric parameters, Table S3: The gradient elution program for CA-Cys, Table S4: The chromatographic separation program.
