# Supplementary material for: Identification and Antioxidant Characterization of Caffeic Acid–Cysteine Adduct in Meat Products Supplemented with Dandelion Extract
Source: Foods. 2026 May 17;15(10):1770. doi: 10.3390/foods15101770 (PMC13205189; doi:10.3390/foods15101770)
Supplement: Supplementary file 1 [file foods-15-01770-s001.zip › foods-4302427-supplementary.pdf]

Supplementary materials

Identification and antioxidant characterization of caffeic acid-cysteine adduct in meat products supplemented with dandelion extract

Xiaohan Li[1], Fengtao Xiang[1], Shaobing Ye[1], Yinhong Chen[2], Hao Sun[2], and Changbo Tang[1,*]

1 State Key Laboratory of Meat Quality Control and Cultured Meat Development, Key Laboratory of Meat Processing, Ministry of Agriculture, Key Lab of Meat Processing and Quality Control, Ministry of Education, Jiangsu Collaborative Innovation Center of Meat Production and Processing, College of Food Science and Technology, Nanjing Agricultural University, Nanjing, 210095, China

2 College of Sciences, Nanjing Agricultural University, Nanjing 210095, China

*Corresponding authors: Dr. Changbo Tang

*E-mail: changbotang@hotmail.com

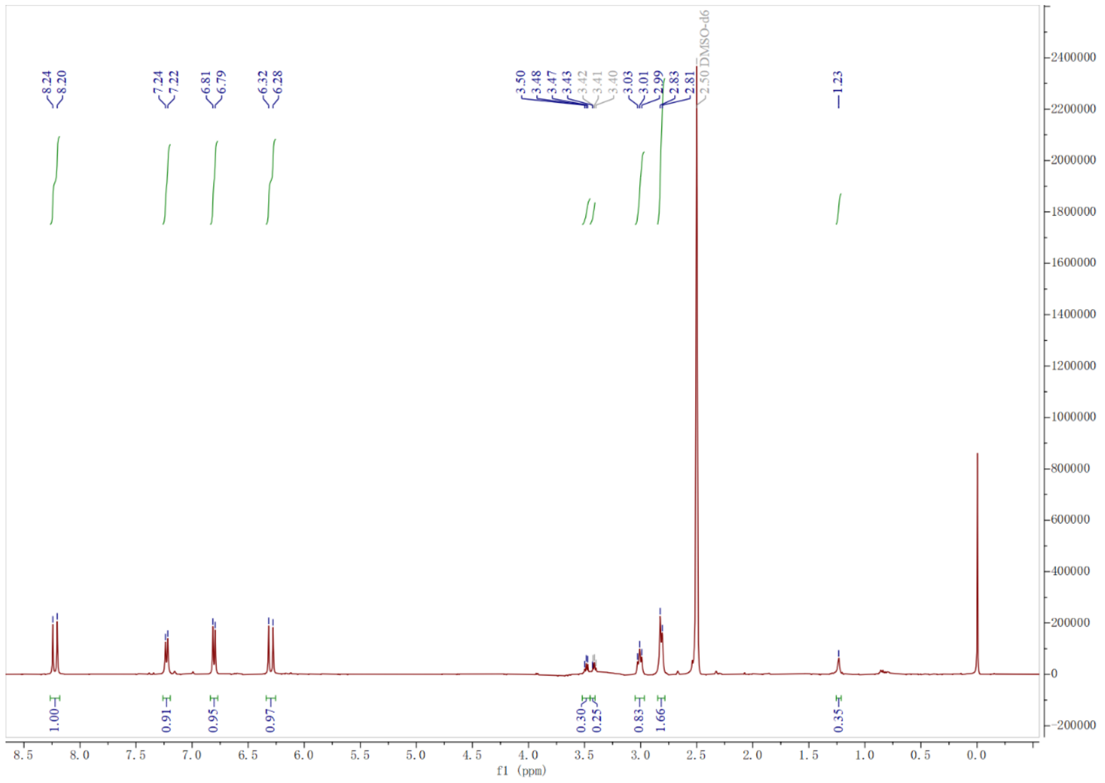

Figure S1 ^{1}H NMR spectrum of CA-Cys.

Table S1 The gradient elution program for dandelion extract.

Time (min)	Mobile phase A (%) 0.1 % formic acid-water	Mobile phase B (%) 0.1 % formic acid- acetonitrile
0.00	99.0	1.0
0.50	99.0	1.0
11.00	60.0	40.0
21.00	1.0	99.0
22.00	1.0	99.0
23.00	99.0	1.0
25.00	99.0	1.0
HSS T3 column	2.1 × 100 mm, 1.8 μm (Waters, USA)	
Column temperature	40°C	
Flow rate	0.4 mL/min	
Injection volume	2 μL	

Table S2 The mass spectrometric parameters.

Parameter	Value
spray voltage	5500 V
temperature	550 °C
declustering potential	80 V
collision energy	35 V

Table S3 The gradient elution program for CA-Cys.

Time (min)	Mobile phase A (%) water	Mobile phase B (%) methanol
0.00	95.0	5.0
0.50	95.0	5.0
12.00	20.0	80.0
13.00	20.0	80.0
15.00	5.0	95.0
17.00	5.0	95.0
17.01	95.0	5.0
20.00	95.0	5.0
BEH C18 column	2.1 × 100 mm, 1.7 µm (Waters, USA)	
Column temperature	40°C	
Flow rate	0.25 mL/min	
Injection volume	5 µL	

Table S4 The chromatographic separation program.

Time (min)	Mobile phase A (%) 0.05% formic acid-water	Mobile phase B (%) methanol
0.00	95.0	5.0
1.00	95.0	5.0
4.00	5.0	95.0
5.40	5.0	95.0
5.60	95.0	5.0
7.50	95.0	5.0
HSS T3 column	2.1 × 100 mm, 1.8 µm (Waters, USA)	
Column temperature	40°C	
Flow rate	0.4 mL/min	
Injection volume	2 µL	
